# Numerical study on the uniform distribution of flow field of airflow dryer

**DOI:** 10.1016/j.heliyon.2024.e29439

**Published:** 2024-04-09

**Authors:** Guanglu Yang, Xuehui Yang, Chunsong Li, Xinfeng Wei, Zhongpu Lu, Chu-an Zhang, Qunlong Wang, Xuehong Wu

**Affiliations:** a School of Energy and Power Engineering, Zhengzhou University of Light Industry, Zhengzhou, 450002, China; b Nanyang Cigarette Factory, China Tobacco Henan Industrial Co., Ltd., Nanyang, 473001, China; c Wuhan Cigarette Factory, China Tobacco Hubei Industrial Co., Ltd., Wuhan, 430051, China

**Keywords:** Tobacco airflow dryer, Deflector, Flow field, Non-uniformity coefficient

## Abstract

The uniformity of hot air flow inside the airflow dryer not only affects the moisture distribution at the outlet, but also affects the quality of the product. Based on the guide plate structure of the SH23A airflow tobacco dryer, a gradient curved guide plate dryer is designed, and the flow field distribution of the dryer is numerically investigated under different flow distribution conditions at the hot air inlet and flue gas inlet. The results show that the airflow uniformity is affected by the flow distribution at the inlet of the heated air and the inlet of the cigarette smoke, the structure of the guide plate, etc., the non-uniformity coefficient decreases with the increase of hot air inlet flow rate. The non-uniformity coefficient of tapered arc deflector decreases by 9–12 %.

## 1 Introduction

In the tobacco processing, the tobacco drying process is an important link in the tobacco silk-making process. Its main work is to dry the cut tobacco and dehydrate the cut tobacco so as to reduce the moisture content of the cut tobacco, so as to meet the requirements of technical standards for cigarette trademarks [1].It can not only enhance the filling and processing resistance of the leaf filaments, but also highlight the flavor style of cigarettes, improve the sensory comfort, so that the sensory quality of tobacco leaves and physical quality to achieve harmony and unity, so as to have a significant impact on the sensory quality of cigarettes. At present, there are main drying methods for tobacco: pneumatic conveying dryers, tunnel dryers, conveyor belt dryers, fixed-bed dryers, spout-fluidized-bed dryers and rotary dryers [2]. Different drying methods have great differences in the treatment strength of tobacco, and the drying effect is completely different. When the tobacco silk is dried by the cylinder dryer the temperature is relatively low, usually lower than 150 °C, the drying time is relatively long, usually 3–4 min, the moisture content of the tobacco silk after drying is relatively stable, which can retain the original aroma of the tobacco well, but because the filling amount is relatively small, it is easy to appear dry head and dry tail [3]. Air flow dryer [4]adopts high temperature and rapid drying method, its drying temperature is higher, usually more than 200 °C, but drying time is shorter, usually only 1–2 s. Compared with drum drying, air flow drying has advantages such as fast speed, high efficiency, low energy consumption, and easy control and maintenance. However, there is an issue of uneven airflow distribution in the air flow dryer. Due to the uneven distribution of leaf fibers during high-speed flow through the pipeline, the heating during drying is uneven. The moisture content of the dried leaves fluctuates greatly, making them prone to clumping and insufficient heating, which can affect the taste of the dried silk product. It may be too dry or too wet, affecting the consistency of the product taste. Therefore, studying the non-uniformity of the flow field inside the airflow dryer, improving the uniformity of the airflow, and ensuring the uniform distribution of temperature and humidity in various parts are the key to improving its production efficiency.

Numerical calculation is an effective method to analyze airflow uniformity, which has been widely used in different industries in recent years [[5], [6], [7], [8], [9], [10]]. Hernandez et al. [11] carried out numerical simulation research on the instability phenomenon in the countercurrent drying process, and identified the difference between the swirl and the free vortex by the strength of the three critical vortices, that is, the reflux zone at the bottom of the dry zone was avoided, while the top of the tower was not affected. El-Mesery et al. [12] evaluated three different airflow patterns: vertical and two horizontal (HMP-1 and HMP-2) inside convective hot air dryers. The minimum specific energy consumption of the horizontal (HMP-2) dryer was found to be 27.44 - MJ kg^-1^, which is 53.82 % lower than the horizontal (HMP-1) dryer and 141.91 % lower than the vertical dryer, and can effectively improve the drying efficiency. Yu et al. [13] adopted the orthogonal experiment method to analyze the influence of these parameters on the drying effect by changing the size of the outlet, changing the spacing of the baking pan and the size of the air speed of the inlet, and obtained the best design parameters of the wire dryer within the experimental range. Liao et al. [14] proposed an MSR-RBF-ARX model for drum tobacco dryer to simulate the drying characteristics of tobacco particles, and found that the contact part between the inner wall of the tower and the outlet end was in an outward form, which was conducive to improving the uniformity of the outlet water, but the overlap of the orifice plates did not affect the uniformity of the outlet water. Olejnik et al. [15] simulated the drying chamber, realized the visualization and optimization of air flow in the drying chamber, and modified the tower air flow dryer by using a rotating disc installed at the bottom of the drying zone, so that the air flow distribution in the dryer was more uniform.

At present, scholars pay more attention to the uniformity and state of the air flow at the inlet of the air flow dryer, but there is still a lack of research on the regulation and non-uniformity of the air flow direction inside the air flow dryer. Focuses on the phenomenon of uneven airflow during the drying process of an airflow dryer. Starting from the perspective of regulating the uniformity of airflow inside the dryer, a two-dimensional model of a conventional guide plate and a gradient arc guide plate structure dryer is constructed. Numerical simulation methods are used to study the uneven distribution of flow field and velocity in each part of the dryer under different airflow distribution conditions, providing a theoretical basis for improving the structure of the drying device.

## 2 Airflow dryer model

### 2.1 Physical model and computational methods

**Fig. 1:**
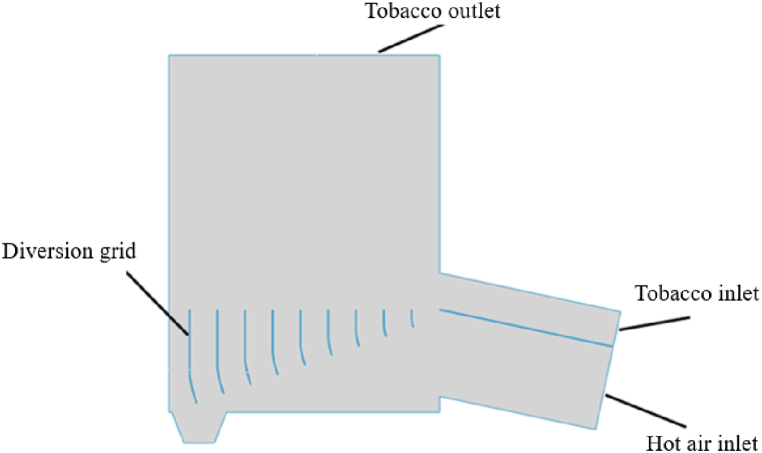
Model of deflector.

**Fig. 2:**
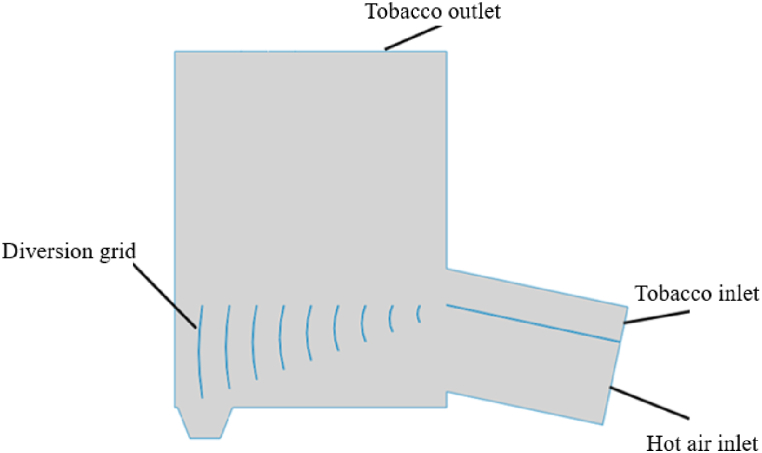
Model of gradual curved deflector.

As shown in Fig. 1 and Fig. 2, two deflector structure dryer models are established: conventional deflector and gradient curved deflector. Under the influence of high-speed air flow, the tobacco silk flows into the feed port at a low place. Under the action of the deflector, the high-speed air passing through the inlet distributes the air evenly inside the dryer and guides it to the dryer so that it continues upward until it reaches the discharge port. To achieve the aim of drying. The thickness of the deflectors of both structures is 6 mm. The length and spacing of the conventional deflectors are shown in Table 1; the arc and length of the gradient arc deflectors are shown in Table 2; the five flow distribution modes are listed in Table 3.

**Table 1:** Details of deflector.

ID	1	2	3	4	5	6	7	8	9
Altitude	114.08	178.15	242.23	306.31	370.38	434.46	498.53	562.16	626.69
Interval	183.5	183.5	183.5	183.5	183.5	183.5	183.5	183.5	183.5

**Table 2:** Details of gradual curved deflector.

编号	1	2	3	4	5	6	7	8	9
弧度	50°	45°	40°	35°	30°	25°	20°	15°	10°
高度	114.08	178.15	242.23	306.31	370.38	434.46	498.53	562.16	626.69
间距	183.5	183.5	183.5	183.5	183.5	183.5	183.5	183.5	183.5

**Table 3:** Traffic distribution table.

Argument	Total flow (m^3^/h)	Hot air flow (m^3^/h)	Tobacco inlet flow (m^3^/h)
Number	58000	30000 35000 40000 45000 50000	28000 23000 18000 13000 8000

In order to simplify the calculation model and improve the calculation speed, the following reasonable assumptions are made: the gap between the guide plate and the shell is ignored in the geometric model; The shell wall of the dryer is smooth and does not exchange heat with the outside world.

### 2.2 Grid independence verification

**Fig. 3:**
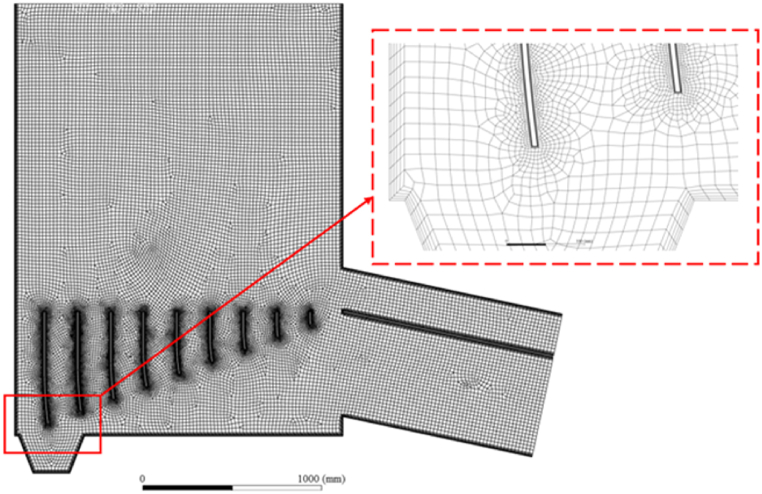
Grid division of conventional flow grid dryer model.

To ensure the accuracy of numerical simulation results and reduce errors in numerical simulation, a five layer grid division is adopted for the outer wall and guide plate of the drying machine, as shown in Fig. 3.

**Fig. 4:**
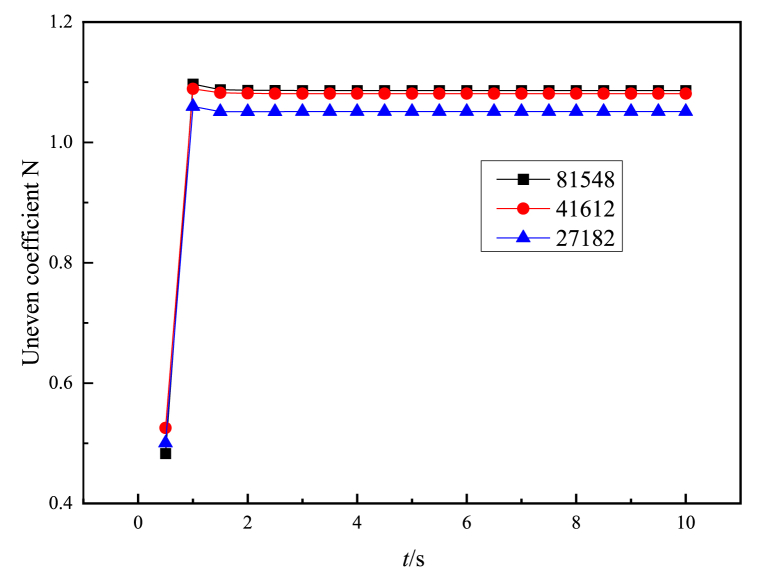
Grid independence verification.

In order to verify the independence of grid quantity, simulations are conducted on drying machines with different grid quantities by adjusting the grid size. The airflow non-uniformity coefficient is selected as the analysis standard, and three sets of grid models with grid quantities of 27182, 41612, and 81548 are simulated. In Fig. 4, it can be seen that as the number of grids increases, the airflow non-uniformity coefficient increases. When the number of grids increases to 41612, the airflow non-uniformity coefficient of the dryer hardly changes with the change of the number of grids. Therefore, 41612 grids are selected for simulation.

### 2.3 Mathematical model

(1) Governing equation

The numerical calculation process is an unsteady turbulent process, which is described by RNG k-ε two-equation turbulence model based on Reynolds' mean equation. The governing equations of the flow model are shown in equation (1) -(4):

(1) $$\frac{\partial \rho }{\partial t}+\frac{\partial }{\partial x_{i}}\left(\rho u_{i}\right)=0,i=1,2,3$$

(2) $$\frac{\partial }{\partial t}\left(\rho u_{i}\right)+\frac{\partial }{\partial x_{j}}\left(\rho u_{i}u_{j}\right)=-\frac{\partial p}{\partial x_{i}}+\frac{\partial \tau_{ij}}{\partial x_{j}},i,j=1,2,3$$

(3) $$\frac{\partial }{\partial t}\left(\rho k\right)+\frac{\partial }{\partial x_{i}}\left(\rho ku_{i}\right)=\frac{\partial }{\partial x_{j}}\left(\alpha_{k}\mu_{t}\frac{\partial k}{\partial x_{j}}\right)+G_{k}+G_{b}-\rho \varepsilon -Y_{M},i,j=1,2,3$$

(4) $$\frac{\partial }{\partial t}\left(\rho \varepsilon \right)+\frac{\partial }{\partial x_{i}}\left(\rho \varepsilon u_{i}\right)=\frac{\partial }{\partial x_{j}}\left(\alpha_{\varepsilon }\mu_{t}\frac{\partial \varepsilon }{\partial x_{j}}\right)+C_{1\varepsilon }\frac{\varepsilon }{k}\left(G_{k}+C_{3\varepsilon }G_{b}\right)-C_{2\varepsilon }\rho \frac{\varepsilon^{2}}{k}-R_{\varepsilon },i,j=1,2,3$$

In the equation, ρ、 $u_{i}$ 、 p 、 k、 ε and $\tau_{ij}$ represent the density, velocity component, pressure, turbulent kinetic energy, turbulent dissipation rate and Reynolds stress, respectively. The remaining parameters and constants are listed in equation (5).

(5) $$\left\{ \begin{array}{c} {\left(\tau_{ij}\right)}_{t}=-\rho \bar{u_{i}^{\prime }u_{j}^{\prime }}=\mu_{t}\left(\frac{\partial u_{i}}{\partial x_{j}}+\frac{\partial u_{j}}{\partial x_{i}}\right)-\frac{2}{3}\left(\rho k+\mu_{t}\frac{\partial u_{k}}{\partial x_{k}}\right)\delta_{ij}\text{,}\\ \mu_{t}=C_{\mu }\frac{\rho k^{2}}{\varepsilon },G_{k}=-\rho \bar{u_{i}^{\prime }u_{j}^{\prime }}\frac{\partial u_{j}}{\partial x_{i}},G_{b}=-g_{i}\frac{\mu_{t}}{\rho \;{\mathrm{Pr}}_{t}}\frac{\partial \rho }{\partial x_{i}}\text{,}\\ Y_{M}=2\rho \varepsilon \frac{k}{a^{2}},R_{\varepsilon }=\frac{C_{\mu }\rho \eta^{3}\left(1-\eta /4.38\right)}{1+0.012\eta^{3}}\frac{\varepsilon^{2}}{k},\eta =\frac{k}{\varepsilon }\sqrt{\frac{1}{2}{\left(\frac{\partial u_{i}}{\partial x_{j}}-\frac{\partial u_{j}}{\partial x_{i}}\right)}^{2}}\text{,}\\ \alpha_{k}=\alpha_{\varepsilon }=1.393,C_{\mu }=0.0845,C_{1\varepsilon }=1.42,C_{2\varepsilon }=1.68,C_{3\varepsilon }=\tanh\left|v/u\right| \end{array}\right.$$

The energy governing equations of convective heat transfer and heat conduction in the whole physical process are shown in equation (6).

(6) $$\frac{\partial }{\partial t}\left(\rho c_{p}T\right)+\frac{\partial }{\partial x_{j}}\left(\rho c_{p}Tu_{j}\right)=\frac{\partial }{\partial x_{j}}\left(\lambda \frac{\partial T}{\partial x_{j}}+u_{k}\tau_{kj}\right),j,k=1,2,3$$

(2) Solve the Settings

The discretization method for the unsteady term of the equation adopts the second-order implicit scheme, the discretization method for the diffusion term adopts the second-order central difference scheme, the discretization method for the density equation, turbulent kinetic energy equation and turbulent dissipation rate equation adopts the second-order upwind scheme, the momentum equation and energy equation adopts the second-order upwind scheme, and the pressure is set to the standard atmospheric pressure. In the vertical direction, the acceleration of gravity is set to −9.81 m/s^2^, and the convergence accuracy is set to 1 × 10^−8^. The boundary conditions and parameters are shown in Table 4.

**Table 4:** Boundary conditions and parameters.

Boundary conditions	parameters	Boundary conditions	parameters
Inlet temperature/°C	200	Outlet pressure/Pa	101325
Convective heat transfer coefficient/W/(m^2^·K)	5	Ambient temperature/°C	25

## 3 Computational results and discussions

### 3.1 Analysis of non-uniformity of airflow

**Fig. 5:**
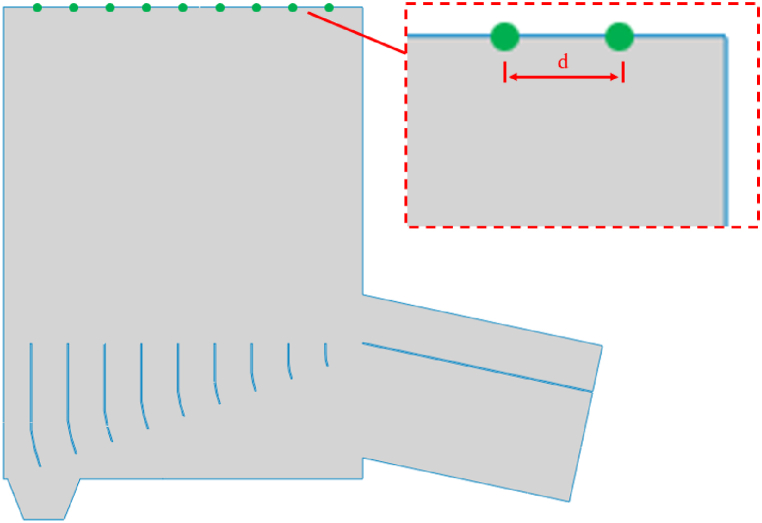
Location arrangement of monitoring points.

The monitoring surface in Fig. 5 is selected to calculate the non-uniformity coefficient of the air flow in the vertical direction of the outlet. There is 9 monitoring points on the monitoring surface, which is evenly distributed, and each monitoring point was separated by 183.5 mm. The coefficient of vertical velocity distribution heterogeneity N is an evaluation index used to quantitatively reflect the degree of vertical velocity distribution heterogeneity. It is calculated according to equation (7):

(7) $$N=\frac{1}{v_{y}}\sqrt{\frac{\sum_{i=1}^{n}{\left(v_{yi}-v_{y}\right)}^{2}}{n}}$$

In the equation, N is the non-uniform velocity coefficient in the vertical direction (y direction) of the monitoring surface, v_y_ is the average vertical velocity (m/s) of all nodes on the monitoring surface, v_yi_ is the vertical velocity (m/s) of all nodes on the monitoring surface, and n is the number of monitoring points on the monitoring surface. The smaller the velocity distribution coefficient is, the more uniform the vertical velocity distribution is.

**Fig. 6:**
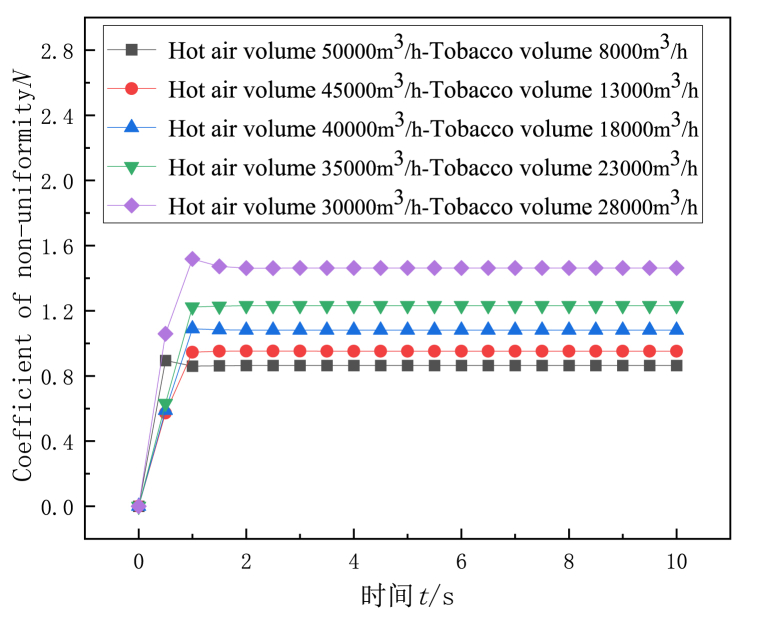
Variation of non-uniformity coefficient of flow velocity on monitoring surface of conventional gate dryer with drying time.

**Fig. 7:**
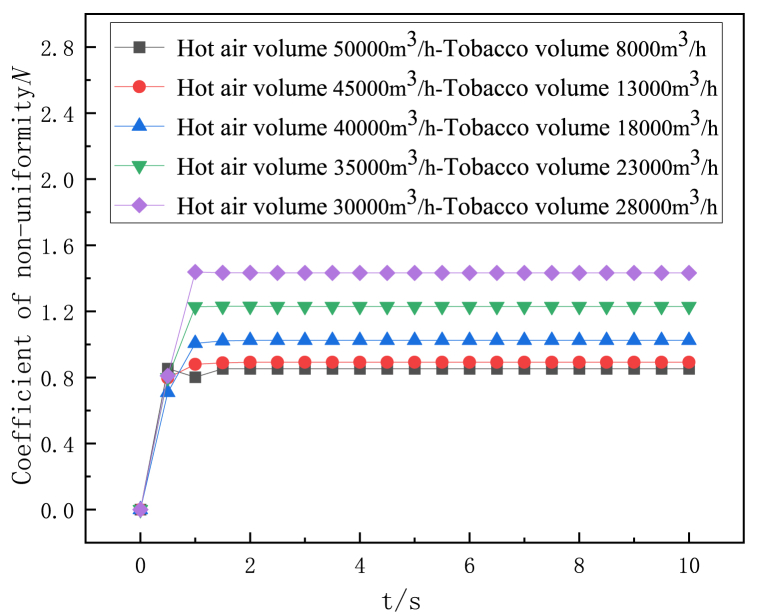
Variation of velocity non-uniformity coefficient of the monitoring surface of the curved deflector dryer with drying time.

Under different flow distribution at hot air inlet and tobacco inlet, the variation trend of the non-uniformity coefficient of the monitoring surface in the dryers with conventional deflector and gradual curved deflector structure along with drying time is shown in Fig. 6 and Fig. 7. It can be seen from the Figure that the non-uniformity of flow rate of the two dryers with deflector structure increases with the decrease of the flow rate at the hot air inlet. Conventional deflector dryer maximum hot air flow rate (hot air inlet air volume 50000 m^3^/h tobacco inlet air volume 8000 m^3^/h) than the lowest hot air flow rate (hot air inlet air volume 30000 m^3^/h tobacco inlet air volume 28000 m^3^/h) of the air non-uniformity coefficient is reduced by 41 %, The airflow non-uniformity coefficient of the highest hot air flow rate of the gradual arc deflector dryer is reduced by 40 % compared with the lowest hot air flow rate. Therefore, properly increasing the flow rate of hot air inlet can improve the uniformity of the flow field in the drying room.

**Fig. 8:**
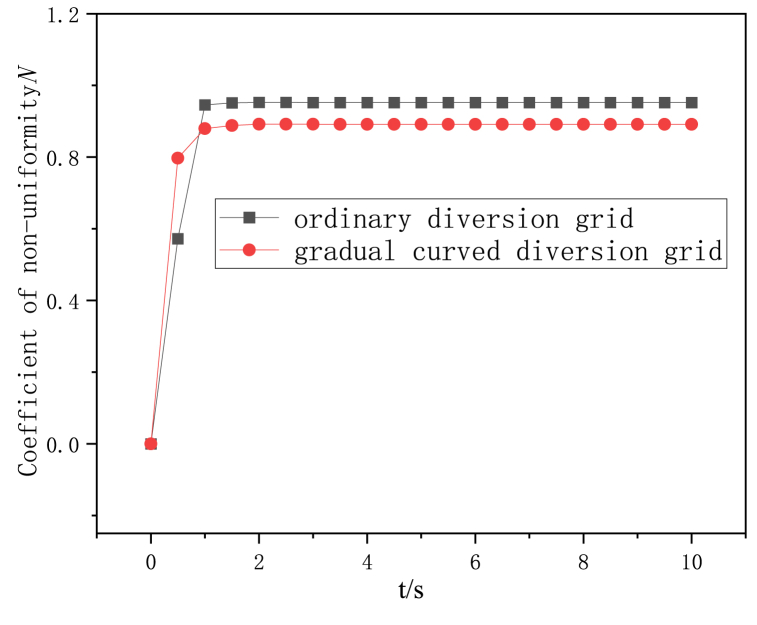
Variation trend of non-uniformity coefficient of the two diversion grids under the hot air flow rate of 50000 m3/h.

Under the condition of high hot air flow (hot air inlet air volume 50000 m^3^/h - tobacco inlet air volume 8000 m^3^/h), the variation trend of the non-uniformity coefficient of the conventional and gradual arc deflector is shown in Fig. 8. It can be seen from the Figure that the non-uniformity coefficient of the two structural deflectors reaches stability at 1s. Compared with the conventional deflectors, the outlet flow uniformity of the gradual arc deflectors is better, and the overall air flow non-uniformity coefficient is reduced by 9–12 %.

### 3.2 Flow field and temperature field analysis

**Fig. 9:**
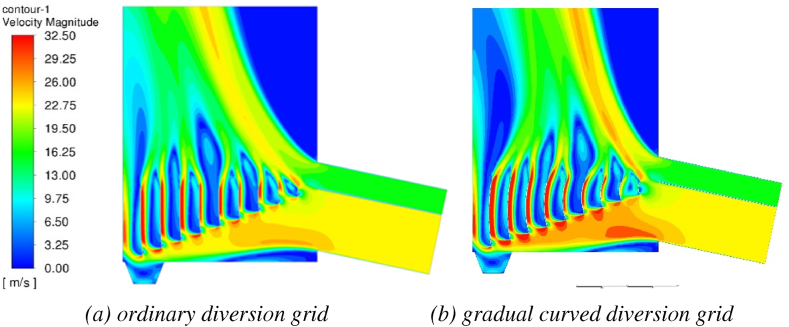
Velocity cloud picture of two kinds of diversion grid (10s).

**Fig. 10:**
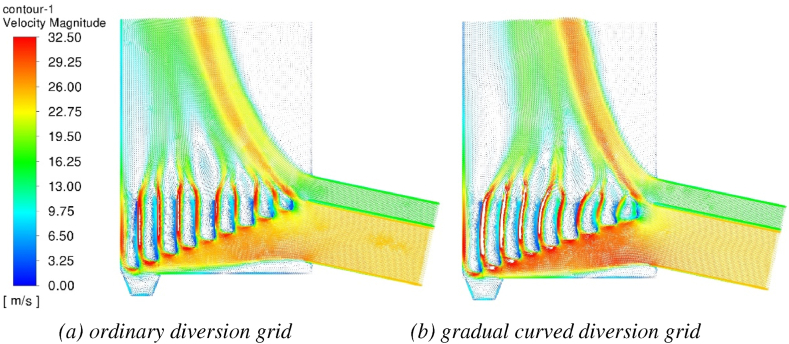
Velocity flow diagram of two kinds of diversion grid (10s).

Under the high flow rate of hot air inlet (hot air inlet air volume 50000 m^3^/h - tobacco inlet air volume 8000 m m^3^/h) of the dryer with conventional deflector and gradual curved deflector structure, the speed distribution cloud diagram of the dryer at 10s is shown in Fig. 9(a and b) and Fig. 10(a and b). It can be seen from the figure that the dryer is affected by the position of the tobacco inlet and the hot air inlet. The whole air flow will tilt to the left wall of the tower body, which will cause the tobacco to gather on the left wall of the drying machine, resulting in uneven drying water and uneven drying of the tobacco. The gradual arc deflector dryer changes the direction of the air flow through the deflector through the arc of the plate body, and hedges with the airflow at the inlet of the tobacco. The right deflector has a larger arc and a larger airflow offset, and the airflow at the inlet of the tobacco is hedged to prevent the airflow from tilting to the left. The left deflector has a smaller arc and little airflow offset, and the flow rate is overall concentrated in the middle of the outlet, and the airflow is more uniform.

The velocity flow diagram of the two deflector dryers is shown in Fig. 10(a and b). It can be seen from the figure that both dryers of the two structures have airflow vortices in the drying chamber, which has an adverse effect on the drying of tobacco. Compared with the airflow of the gradual curved deflector structure, the conventional deflector has more airflow vortices, and the airflow is more uneven. The main reason is that due to the influence of the inlet air volume and inlet position, the conventional deflector air flow to the left leads to a large flow dead zone above the inlet.

**Fig. 11:**
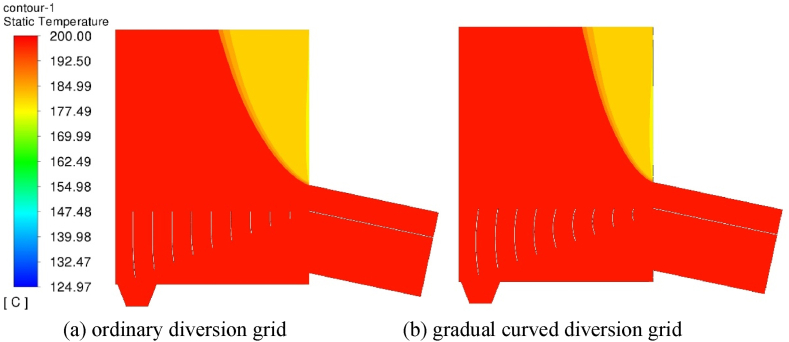
Temperature distribution of two kinds of diversion grid (10s).

The temperature distribution of the dryer with two structures is shown in Fig. 11(a, b). As can be seen from the Figure, there is no change in the air flow temperature in the wire dryer when no tobacco is dried, and the flow field distribution will also lead to changes in the temperature field in the wire dryer. Because there is a large flow dead zone above the hot air inlet of the conventional deflector, a larger area of low temperature area is shown in the temperature field.

## 4 Conclusion

The effect of the distribution of hot air inlet and tobacco inlet air volume on the uniformity of flow field in conventional and gradual arc deflector dryers is studied numerically, and the following conclusions can be drawn.

(1) In the flow range of the study, the non-uniformity of the flow rate of the two deflector structure dryers increases with the decrease of the hot air inlet flow rate, and the non-uniformity coefficient of the maximum hot air flow rate of the conventional deflector dryer is reduced by 41 % compared with the lowest hot air flow rate. The airflow non-uniformity coefficient of the highest hot air flow rate of the gradual arc deflector dryer is reduced by 40 % compared with the lowest hot air flow rate.
(2) The non-uniformity coefficient of the two structures of the deflector is stable before 1s. Compared with the conventional deflector, the outlet flow uniformity of the gradient arc deflector is better, and the overall air flow non-uniformity coefficient is reduced by 9–12 %.
(3) There are more vortex areas in the air flow of the conventional deflector than that of the gradual arc deflector structure, and the air flow is more uneven.

## CRediT authorship contribution statement

**Guanglu Yang:** Software, Data curation. **Xuehui Yang:** Methodology, Investigation, Data curation. **Chunsong Li:** Visualization, Validation, Software. **Xinfeng Wei:** Visualization, Validation, Software, Methodology, Investigation. **Zhongpu Lu:** Writing – original draft, Visualization, Software. **Chu-an Zhang:** Writing – review & editing, Conceptualization. **Qunlong Wang:** Writing – original draft, Validation, Data curation. **Xuehong Wu:** Writing – review & editing, Writing – original draft, Supervision, Methodology.

## Declaration of competing interest

The authors declare the following financial interests/personal relationships which may be considered as potential competing interests: Xuehong Wu reports financial support was provided by 10.13039/501100004262Zhengzhou University of Light Industry. If there are other authors, they declare that they have no known competing financial interests or personal relationships that could have appeared to influence the work reported in this paper.
